# Chromosomal mapping of microsatellite repeats in the rock bream fish *Oplegnathus fasciatus*, with emphasis of their distribution in the neo-Y chromosome

**DOI:** 10.1186/1755-8166-6-12

**Published:** 2013-03-19

**Authors:** Dongdong Xu, Bao Lou, Luiz Antonio Carlos Bertollo, Marcelo de Bello Cioffi

**Affiliations:** 1 Key Lab of Mariculture and Enhancement of Zhejiang Province, Marine Fishery Institute of Zhejiang Province, Zhoushan, 316100, P. R. China; 2 Marine and Fishery Institute, Zhejiang Ocean University, Zhoushan, Zhejiang Province, 316100, P. R. China; 3 Departamento de Genética e Evolução, Universidade Federal de São Carlos, São Carlos, SP, Brazil

**Keywords:** Oplegnatidae, X_1_X_2_Y sex chromosomes, Fluorescence *in situ* hybridization, Repetitive DNAs, Chromosomal differentiation

## Abstract

Despite the theoretical and experimental progress, our understanding on sex chromosome differentiation is still diagrammatic. The accumulation of repetitive DNA sequences is believed to occur in early stages of such differentiation. As fish species present a wide range of sex chromosome systems they are excellent models to examine the differentiation of these chromosomes. In the present study, the chromosomal distribution of 9 mono-, di- and tri-nucleotide microsatellites were analyzed using fluorescence *in situ* hybrization (FISH) in rock bream fish (*Oplegnathus fasciatus*), which is characterized by an X_1_X_2_Y sex chromosome system. Generally, the males and females exhibited the same autosomal pattern of distribution for a specific microsatellite probe. The male specific Y chromosome displays a specific amount of distinct microsatellites repeats along both arms. However, the accumulation of these repetitive sequences was not accompanied by a huge heterochromatinization process. The present data provide new insights into the chromosomal constitution of the multiple sex chromosomes and allow further investigations on the true role of the microsatellite repeats in the differentiation process of this sex system.

## Background

The origin and evolution of sex chromosomes are among the most interesting topics in evolutionary genetics. Although sex chromosomes evolve from a homologue pair of autosomes, over time they become different, both from each other and the autosomes, in gene content and structure [1,2]. The processes working on sex chromosome differentiation are still not completely understood. However, the accumulation of repetitive DNA sequences is one of the first probable steps in the early stages of such differentiation [2-4]. Repetitive sequences, which can account for more than 50% of the genome, constitute the substantial portion of eukaryotic genomes and include the tandem repeats (satellites, minisatellites and microsatellites) and dispersed elements (transposons and retrotransposons) (reviewed in [5]). A clear correlation between sex chromosomes and repetitive DNAs has been evidenced by a number of studies [4,6-11], suggesting that the differentiation of sex chromosomes is frequently associated with the accumulation of such repetitive sequences. The chromosomal mapping of repetitive DNAs has provided new insights for understanding genome evolution and was useful to reveal the process of sex chromosome differentiation in many vertebrate species.

Teleost fishes are an outstanding model to study the evolution of sex chromosome since they present a broad range of sex chromosome systems, as well as the absence of differentiated sex chromosomes in most species [12,13]. Besides, they have much younger sex chromosomes compared to higher vertebrates, such as mammals and birds, making it possible to analyze the early stage of their differentiation [2,14,15]. Particularly, repeated DNA sequences have been applied to clarify the potential role of these sequences in the differentiation of fish chromosomes (reviewed in [16]).

The rock bream (*Oplegnathus fasciatus*) belongs to the Oplegnathidae family and is one of the most economically important marine fish in East Asia [17]. Conventional cytogenetic analysis of this species showed that the male karyotype is composed of 2n = 47 chromosomes (3 m + 44a), while the female karyotype is composed of 2n = 48 chromosomes (2 m + 46a). This species is characterized by having a multiple X_1_X_1_X_2_X_2_/X_1_X_2_Y sex chromosome system [18,19]. To further understanding of sex chromosome differentiation in *Oplegnathus fasciatus*, we mapped the chromosomal distribution of different classes of microsatellite repeats in the genome of *Oplegnathus fasciatus*, focusing on their distribution within the sex chromosomes.

## Results

### Karyotyping

**Figure 1:**
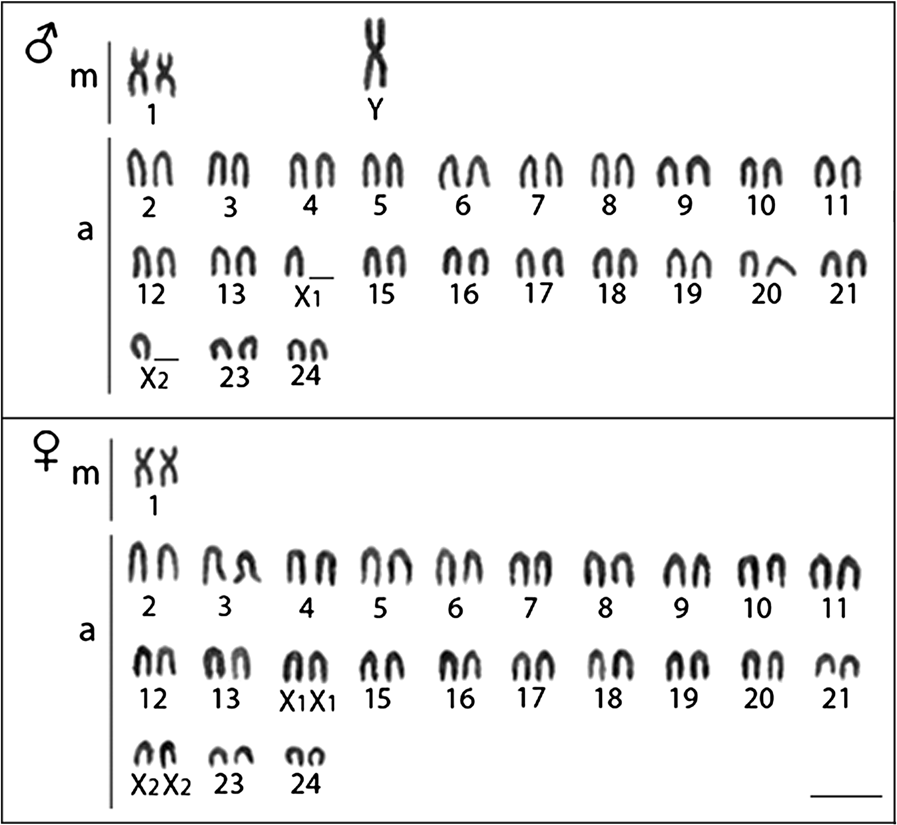
**Giemsa-stained male (above) and female (below) karyotypes of *Oplegnathus fasciatus*, highlighting the presence of an unusual X**_**1**_**X**_**2**_**Y sex chromosome system. Bar = 5 μm.**

*Oplegnathus fasciatus* showed 2n = 48 chromosomes (46a + 2 m) in the female and 2n = 47 chromosomes (44a + 3 m) in the male specimens. This specific sex karyotype is determined by the characteristic multiple sex chromosome system, with X_1_X_1_X_2_X_2_ chromosomes in the females and X_1_X_2_Y chromosomes in the males, where the Y chromosome corresponds to a metacentric one, easily recognized by its larger size compared to the other chromosomes. Differently, the X_1_ and X_2_ chromosomes are acrocentrics and not easily identifiable. Thus, both chromosomes were tentatively located as the 14th and 22nd pairs in the karyotype, respectively (Figure 1).

### Chromosomal mapping of the microsatellite repeats

In general, the same distribution pattern was found between males and females when looking at the mapping of a specific microsatellite probe in the autosomes. A discrete banding pattern was observed for some microsatellites, whereas others were more widely dispersed along the chromosomes. However, the Y chromosome demonstrated a remarkable and specific accumulation of several microsatellites.

**Figure 2:**
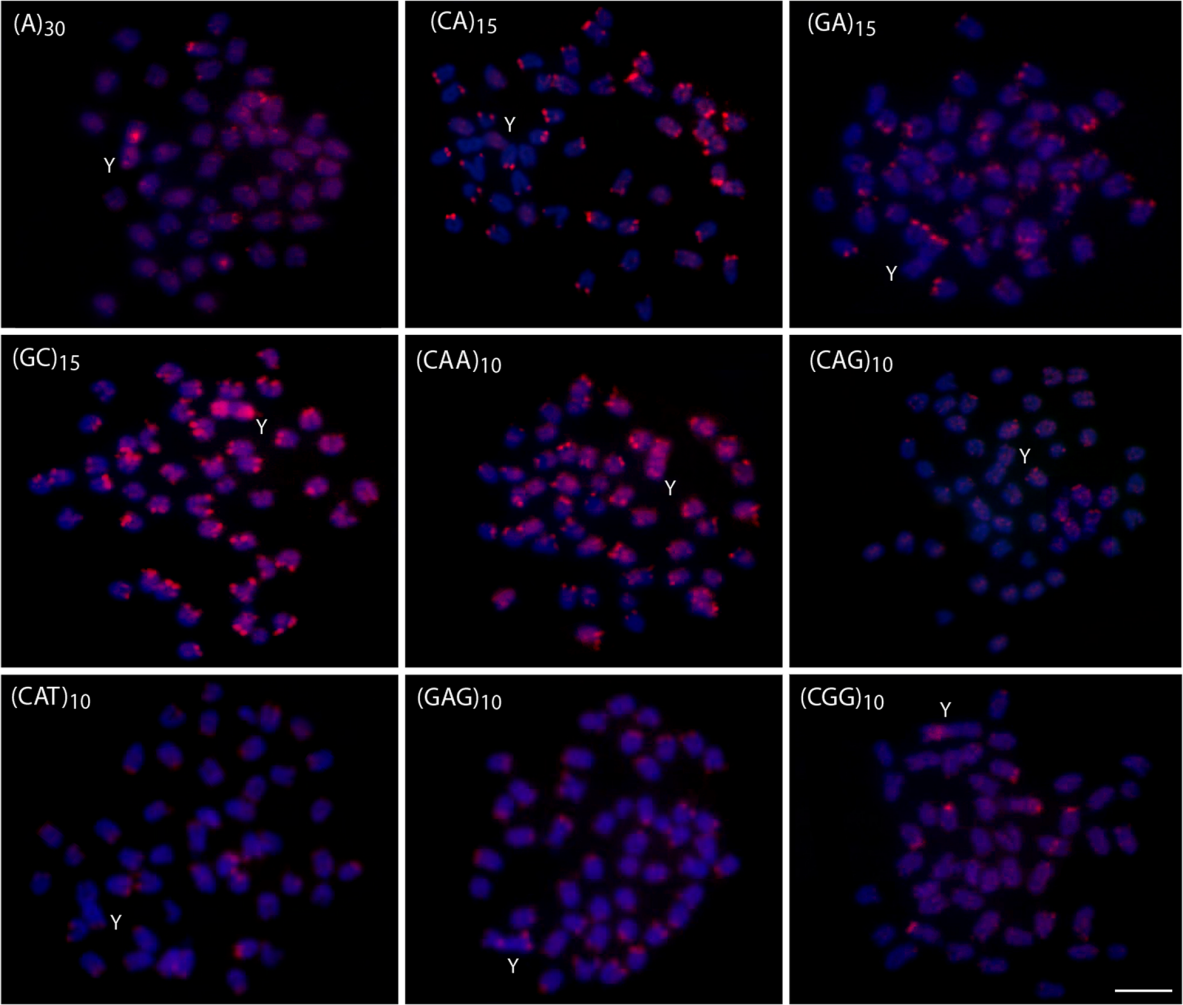
**Mitotic metaphase chromosomes of *Oplegnathus fasciatus *males with an X**_**1**_**X**_**2**_**Y sex chromosome system, hybridized with different labeled microsatellite-containing oligonucleotides.** Chromosomes were counterstained with DAPI (blue) and microsatellites probes were directly labeled with Cy3 during synthesis (red signals). Letters mark the Y chromosomes. Bar = 5 μm.

The microsatellites d(CA)_15_, d(GA)_15_, d(CAT)_10_ and d(GAG)_10_, provided preferential banding pattern in the subtelomeric region along most chromosome arms, with some signals appearing stronger and more extended than the others. The microsatellites d(GC)_15_ and d(CAA)_10_ provided strong dispersed signals across the entire length of most chromosomes, highlighting their widespread presence in the genome of *Oplegnathus fasciatus*. In addition, d(A)_30_ d(CAG)_10_ and d(CGG)_10_ produced also a scattered, but more discrete distribution in the chromosomes (Figure 2).

**Figure 3:**
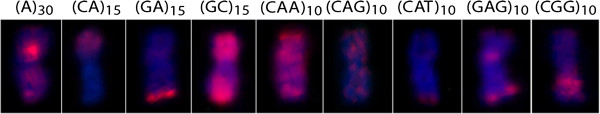
**Y chromosomes of *Oplegnathus fasciatus, *highlighting the patterns of distribution for microsatellites, derived from FISH data.** Note the huge distribution of some classes of microsatellites on this chromosome.

The specific male Y chromosome is easily characterized by a strong concentration of some microsatellite repeats. Particularly the microsatellites d(GC)_15_ and d(CAA)_10_ are highly distributed in the Y chromosome, being practically accumulated along its entire length. A stronger, but less concentrated distribution, was also observed for microsatellites d(A)_30_ and d(CAG)_10_, while the microsatellites d(CA)_15_, d(CGG)_10_, d(GA)_15_, d(CAT)_10_ and (dGAG)_10_ were preferentially clustered on specific regions of the chromosome. Figure 3 highlights the overall distribution of all microsatellites on the Y chromosome.

## Discussion

### Chromosomal distribution of microsatellites on autosomes

Our results were able to evidence that the distribution of the microsatellites in the chromosomes of the rock bream fish differs among the distinct repeats analyzed. Indeed, a strong accumulation occurs for some of them, as is the case of the d(GC)_15_ and d(CAA)_10_ repeats, while others have a distinct and more discrete distribution pattern. Thus, the genome of the rock bream shows a clear differential accumulation of microsatellites along its evolutionary time. Similarly, some of these microsatellites were also found to be clustered in other fish species, such as in the Silurformes *Imparfinis schubarti* (Heptapteridae), *Steindachneridion scripta* (Pimelodidae) and *Rineloricaria latirostris* (Loricariidae), which exhibit a remarkable accumulation of both (GA)_15_ and (A)_30_ microsatellites in the telomeric regions of their chromosomes [20]. In two karyomorphs of *Hoplias malabaricus* ‘species complex’, one of them displaying an XY sex chromosome system and the other one an X_1_X_2_Y multiple system, the (GA)_15_ and (CA)_15_ repeats are preferentially located in the subtelomeric regions [21]. This preferential accumulation in particular locations may indicate chromosomal regions where microsatellites are present as very large perfect or degenerate arrays [4]. However, it is known that microsatellite repeats can also exhibit wide diversity with respect to chromosomal location and distribution [16]. For example, the microsatellites d(GC)_15_ and d(CAA)_10_, which provided the strongest dispersed signals across the entire length of most chromosomes in rock bream, were found to have a subtelomeric location in *H. malabaricus*[21]. As a whole, these data demonstrate the dynamism in respect to the accumulation and distribution of repetitive DNAs on fish genomes.

### Patterns of microsatellite distribution on Y chromosome

Multiple sex chromosomes in fishes usually arise from centric or tandem fusions between ancestral sex chromosomes [15,22], and repetitive DNA sequences have proven to be useful markers of these processes. This is the case for d(GAG)_10_ repeats in the present study. Indeed, this microsatellite showed a general location in the telomeric region of the chromosomes (Figure 2). In addition, this same microsatellite provided a clear banding pattern not only in the telomeric regions of the Y chromosome, but also on its centromeric region. Whereas this chromosome was originated from a centric fusion that merged the ancestral homologues of the X_1_ and X_2_ chromosomes, this centromeric site is a clear indicator of that chromosomal rearrangement.

On the other hand, other microsatellites have a wide distribution along the Y chromosome, particularly the d(GC)_15_ and d(CAA)_10_, and even the d(A)_30_ d(CAG)_10_ ones. The accumulation of repetitive DNA sequences was likely to play an important role in the differentiation process of sex chromosomes, especially XY and ZW sex systems [16]. Suppression of recombination is a prerequisite for stable genetically determined sex systems, and thus the massive accumulation of repetitive sequences, including microsatellites, usually occurs in non-recombining regions [2]. In simple sex systems, the repetitive DNAs usually accumulate in the heterochromatic regions, thereby forming heterochromatic block which could drive the divergence of sex chromosomes [2,23,24].

Although the association of repetitive DNA sequences with the differentiation of multiple sex chromosome systems has also been testified, there is no significant increase of heterochromation in such multiple sex chromosomes. For example, in *H. malabaricus* as well as *E. erythrinus*, the multiple sex chromosomes do not display a great amount of heterochromatin [15,25]. Furthermore, the heterochromatin that is present in the sex chromosomes is ‘pre-existing’, with no significant increase on its amount after the differentiation of the multiple systems. These evidences indicated that the differentiation of multiple sex system is achieved through chromosomal rearrangements that occurred during their own origin [21]. Thus, chromosomal rearrangements may create new linkage groups between genes that were originally found in different chromosomes, including sexually antagonistic ones. This may lead to reduced or suppressed recombination in regions close to the breakpoints in the heterozygote [26]. Concerning *O. fasciatus* the conspicuous amount of microsatellite repeats in the Y chromosome is yet an open question which deserves further investigation. As we have not at this moment a clear identification of the X_1_ and X_2_ chromosomes in the karyotype, it is not clear if these repeats were already present in the ancestral chromosomes or if they have been accumulated after the fusion process originating the neo-Y chromosome. In the latter case, we would have a clear example of a huge accumulation of repetitive DNAs on the sex specific chromosome and this would be an interesting novelty relative to a multiple sex chromosome system, keeping also in mind that our previous C-banding data showed that no huge heterochromatic content is presented in the Y chromosomes of *O. fasciatus*[18,19].

## Conclusions

In summary, *O. fasciatus* has a characteristic X_1_X_2_Y sex chromosome system in which the large metacentric Y chromosome displays a specific amount of distinct microsatellites repeats along both arms. In addition, several autosomes also show a conspicuous distribution pattern of these DNA repeats, showing that several classes of repetitive DNA sequences have an important role in the genome differentiation of this species, including the sex chromosomes. However, the accumulation of these repetitive sequences was not accompanied by a huge heterochromatinization process. At the moment, the present data provide new insights into the chromosomal constitution of multiple sex chromosomes and allow further investigations on the true role of the microsatellite repeats in the differentiation process of this sex system.

## Methods

### Specimens, mitotic chromosome preparation, chromosome staining and karyotyping

**Figure 4:**
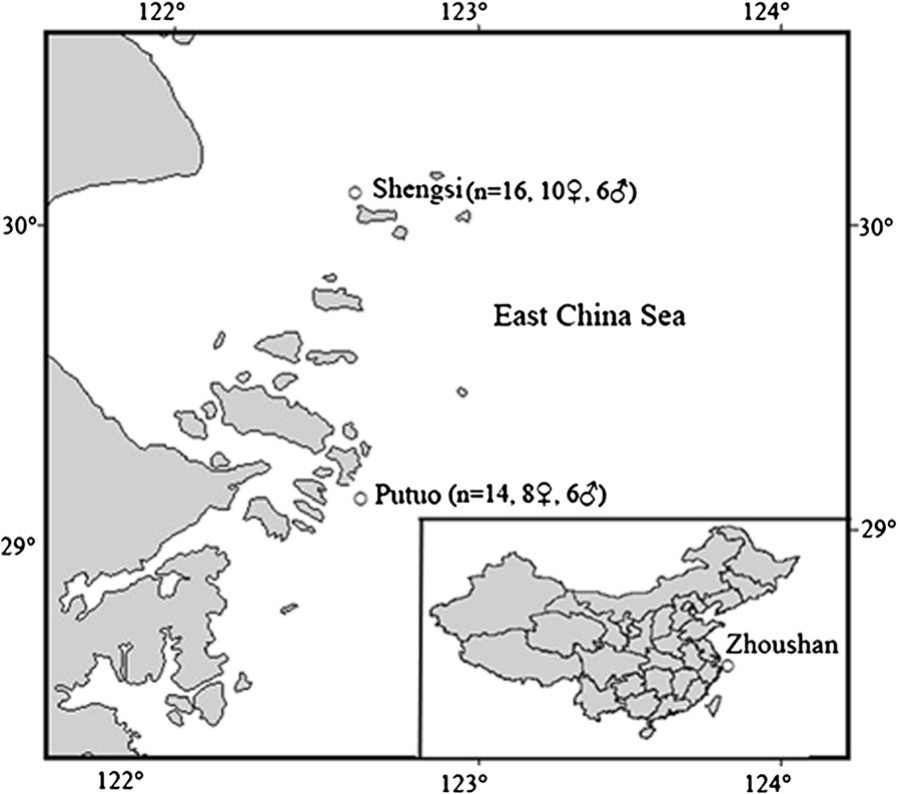
**Map of sampling locations of rock bream along the coasts of Zhoushan, (China).** Fish were collected at Putuo (n = 14) and Shengsi (n = 16). A more detailed geographic definition of the gray areas is highlighted in box.

A total of 30 adult fish (18 females and 12 males), with easily recognizable tests or ovaries, were collected from the coast of Zhoushan (Zhejiang Province) (Figure 4). Mitotic chromosome preparations were obtained by the air-drying method. The specimens were injected with 0.05% colchicine for 3 hours. The kidney tissue was collected and placed in hypotonic 0.075 mol/l KCl solution for 30 minutes, to obtaining a cell suspension. The cells were fixed in Carnoy’s solution (methanol: acetic acid, 3: 1, v/v). Afterwards, the cells were dropped on cooled clean glass slides, air-dried and stained with 15% Giemsa solution diluted with phosphate buffer (pH 6.8).

### Fluorescence in situ hybridization on mitotic spreads

Fluorescence *in situ* hybridization experiments were performed as described in [4] with slight modifications. We used the following labeled oligonucleotides as probes: d(A)_30_, d(CA)_15_, d(GA)_15_, d(GC)_15_, d(CAA)_10_, d(CAG)_10_, d(CAT)_10_, d(GAG)_10_ and d(CGG)_10_. These sequences were directly labeled with Cy3 at 5^′^ terminal during synthesis by Sigma (St. Louis, MO, USA). The chromosomes were counterstained with DAPI (1.2 μg/ml), mounted in antifade solution (Vector, Burlingame, CA, USA), and analyzed in an epifluorescence microscope Olympus BX50 (Olympus Corporation, Ishikawa, Japan).

Approximately 30 metaphase spreads were analyzed per specimen to determine the diploid chromosome number and karyotype structure. The chromosomes were classified as metacentric (m) or acrocentric (a) according to arm ratios [27].

## Abbreviations

2n: Diploid number; a: Acrocentric chromosome; DAPI: 4^′^6-diamidino-2-phenylindole; FISH: Fluorescence *in situ* hybridization; m: Metacentric chromosome

## Competing interests

The authors declare that they have no competing interests.

## Authors’ contributions

XD carried out the conventional cytogenetic analysis, coordinated the study and drafted the manuscript, LB helped in the conventional cytogenetic analysis and drafted the manuscript, LACB drafted and revised the manuscript, MBC carried out the molecular cytogenetic analysis, drafted and revised the manuscript. All authors read and approved the final version of the manuscript.
